# Treatment-Related Toxicities During Anti-GD2 Immunotherapy in High-Risk Neuroblastoma Patients

**DOI:** 10.3389/fonc.2020.601076

**Published:** 2021-02-17

**Authors:** Thomas Blom, Roosmarijn Lurvink, Leonie Aleven, Maarten Mensink, Tom Wolfs, Miranda Dierselhuis, Natasha van Eijkelenburg, Kathelijne Kraal, Max van Noesel, Martine van Grotel, Godelieve Tytgat

**Affiliations:** ^1^Princess Máxima Center for Pediatric Oncology, Utrecht, Netherlands; ^2^Department of Pediatric Infectious Diseases, Wilhelmina Children’s Hospital, University Medical Center Utrecht, Utrecht, Netherlands

**Keywords:** neuroblastoma, immunotherapy, dinutuximab, ch14.18, anti-GD2 antibody, safety, toxicity

## Abstract

The introduction of immunotherapy using an anti-GD2 antibody (dinutuximab, ch14.18) has significantly improved survival rates for high-risk neuroblastoma patients. However, this improvement in survival is accompanied by a substantial immunotherapy-related toxicity burden. The primary objective of this study was to describe treatment-related toxicities during immunotherapy with dinutuximab, IL-2, GM-CSF, and isotretinoin. A retrospective, single center analysis of immunotherapy-related toxicities was performed in twenty-six consecutive high-risk neuroblastoma patients who received immunotherapy as maintenance therapy in the Princess Máxima Center (Utrecht, Netherlands). Toxicities were recorded and graded according to the CTCAE. Particular attention was drawn to pain and fever management and toxicities leading to dose modifications of dinutuximab and IL-2. Twenty-three patients (88%) completed all six courses of immunotherapy. Disease progression, isotretinoin-associated liver toxicity, and catheter-related infection in combination with peripheral neuropathy were reasons for immunotherapy discontinuation. The most common grade ≥3 toxicities for courses 1–5, respectively, were pain, catheter-related infections, and fever. In total, 310 grade ≥3 toxicities were recorded in 124 courses. Thirty-three grade 4 toxicities in 19/26 patients and no grade 5 toxicities (death) were seen. Fifty-nine percent of grade ≥3 toxicities were recorded in the two courses with IL-2. Catheter-related bloodstream infections were identified in 81% of patients. Four of these episodes led to intensive care admission followed by full recovery (grade 4).

## Highlights

Immunotherapy-related toxicities after induction and consolidation according to the Dutch Childhood Oncology Group (DCOG) NBL2009 treatment protocol are considerable but manageable. More toxicity is observed in the immunotherapy courses containing IL-2.

## Introduction

Despite intensive treatment regimens, patients with high-risk neuroblastoma experienced poor survival outcomes (1). The introduction of immunotherapy using a chimeric anti-GD2 monoclonal antibody (dinutuximab, ch14.18) combined with immunostimulatory cytokines [interleukin-2 (IL-2) and granulocyte-macrophage colony-stimulating factor (GM-CSF)] has significantly improved survival rates for these patients (2). The disialoganglioside GD2, has relative tumor-selective expression, with only weak expression in normal human tissues like neurons, melanocytes, and peripheral nerve pain fibers (3), making it an attractive target for neuroblastoma-specific immunotherapy. However, as early as in the first clinical reports, the substantial toxicity burden caused by the ch14.18 antibody was recognized (4, 5), with patients suffering from intense, morphine-responsive pain, intermittent fever, allergic reactions (exanthema, urticaria), and changes in blood pressure. To increase antibody-dependent cellular cytotoxicity (ADCC) and the subsequent antitumor effect, the addition of immunostimulatory cytokines (IL-2, GM-CSF) was studied with encouraging results (6, 7). Here, the clinical benefit has to be weighed against the potential toxicity of these cytokines. Treatment-related toxic effects have resulted in treatment discontinuation and even deaths (2, 8, 9).

From 2016 onwards, high-risk neuroblastoma patients receive immunotherapy as maintenance therapy in the Princess Máxima Center for Pediatric Oncology (Utrecht, The Netherlands). The aim of this study was to describe treatment-related toxicities from immunotherapy with dinutuximab, cytokines IL-2 and GM-CSF, and isotretinoin in a cohort of 26 high-risk neuroblastoma patients treated with induction and consolidation therapy according to the DCOG (Dutch Childhood Oncology Group) NBL2009 high-risk group protocol (10). Specifically, we studied pain and fever management and treatment-related toxicities leading to dose modifications of dinutuximab and IL-2.

Additionally, we performed a non-systematic literature review on toxicity associated with ch14.18 antibody-based immunotherapy in patients with neuroblastoma.

## Materials and Methods

### Patient Population

A retrospective, single center analysis of immunotherapy-related toxicities was performed in twenty-six consecutive high-risk neuroblastoma patients who received dinutuximab-based immunotherapy as maintenance therapy between August 2016 and October 2019 in the Princess Máxima Center for Pediatric Oncology (Utrecht, The Netherlands). The high-risk patient cohort consisted of International Neuroblastoma Risk Group [INRG (11)] stage M and ≥ 12 months at diagnosis, or INRG stage L2 with *MYCN* amplification. All patients had completed induction and consolidation therapy according to the DCOG NBL2009 treatment protocol (10), which is based on the standard arm of the German GPOH (Gesellschaft für Pädiatrische Onkologie und Hämatologie) NB2004 high-risk protocol (12). Patients who achieved at least partial response were eligible to receive immunotherapy. Patients with relapse were not included. Other requirements were Lansky Performance Scale score of ≥60%, adequate organ functions, and full recovery from any toxicities from previous treatments.

### Immunotherapy Protocol

An overview of the six immunotherapy courses is provided in **Supplementary Figure 1**. The first five patients received dinutuximab (ch14.18/SP2/0; United Therapeutics Corporation, USA) under a named-patient program at a dose of 17.5 mg/m^2^ per day as a 10 h (20 h maximum) intravenous infusion on 4 consecutive days. After the approval of dinutuximab beta by the European Medicines Agency (EMA) in May 2017, patients received dinutuximab beta (ch14.18/CHO; EUSA Pharma, Netherlands) at a dose of 20 mg/m² per day as an 8 h (16 h maximum) infusion on 5 consecutive days. During courses 1, 3, and 5, GM-CSF was administered for 14 consecutive days. During courses 2 and 4, IL-2 was administered by continuous intravenous infusion at a dose of 3.0 x 10^6^ and 4.5 x 10^6^ IU/m^2^/day in weeks 1 and 2, respectively. All patients received isotretinoin at a dose of 160 mg/m² per day for 14 days per course. Course 6 solely consisted of isotretinoin.

### Pain Management and Prophylactic Medication

Pain management consisted of oral gabapentin (15 mg/kg/day in three doses) starting 7 days prior to start of dinutuximab infusion, and intravenous acetaminophen (60 mg/kg/day in four doses, with a maximum of 4 g/day) and morphine (10 µg/kg/h) starting 1 and 2 h before the start of dinutuximab infusion, respectively. Gabapentin and morphine were continued during dinutuximab infusion. Individual patients were closely monitored by the pain anesthesiologist. In case of inadequate pain control, a personalized combination of intermittent IV morphine boluses, esketamine (0.1–0.4 mg/kg/h), clonidine (1–6 µg/kg/day), and amitriptyline (0.5–2 mg/kg/day) was used. When morphine was not tolerated due to side effects or renal failure, piritramide was used instead.

Prophylactic treatment for immune-related symptoms with antihistamines consisted of the combination of clemastine, cetirizine, and ranitidine.

### Toxicity

Vital parameters, laboratory results including blood culture results, and other toxicities were prospectively recorded in patients’ medical and nursing files and retrospectively graded according to the National Cancer Institute Common Terminology Criteria for Adverse Events (CTCAE; version 3.0). In case toxicities were not listed in CTCAE version 3.0 (e.g. Cytokine release syndrome), CTCAE version 5.0 was used. Data from grade 1–2 toxicities are not reported, with the exception of fever and grade 1–2 toxicities that resulted in dose modifications of dinutuximab and/or IL-2.

During dinutuximab infusion, pain scores were obtained at least every 4 h. Intensity of pain was assessed using the COMFORT Behavior Scale for patients <3 years of age (13), the Wong–Baker Faces Pain Rating Scale (WB-FPRS) for patients between the age of 3 and 8 (14), and the visual analogue scale (VAS) for children ≥8 years of age (15). COMFORT scores >24 and WB-FPRS and VAS scores ≥7 were considered as severe pain (grade 3). In the case of disabling pain, pain was graded as grade 4.

The maximum body temperature per day was used to assess fever instances. Acetaminophen and diclofenac were, respectively, used as first- and second-line pharmacologic antipyretic therapy. We defined catheter-related infection (CRI) as blood culture-proven bacteremia in association with (1) clinical evidence of infection (fever, tachycardia, hypotension, etc.), (2) no probable other site of infection or cause for bacteremia, and (3) treated with systemic antibiotic therapy. Pediatric intensive care unit (PICU) admission due to a CRI was graded as grade 4. Empirical antibiotic therapy started after fever and discontinued after normalization of symptoms in combination with negative or contaminated blood cultures were not regarded as infections. For this study, blood culture results were reviewed by a pediatric infectious disease physician and categorized based on identification and pathogenicity of isolated bacteria.

### Dose Modifications of Dinutuximab and Interleukin-2

We collected all dose modifications of dinutuximab and IL-2 from medical and nursing files. We differentiated between a 50% decrease in the infusion rate, temporary interruption and complete cessation of dinutuximab/IL-2 infusion. For every dose reduction, the causative toxicity was recorded. To investigate the effect of dinutuximab dose modifications, we calculated the total administered dose per course per patient as percentage of intended dose.

### Statistical Analysis

McNemar’s test for paired data was used to compare the incidence of toxicities between courses containing GM-CSF (courses 1, 3, and 5) and IL-2 (courses 2 and 4). An independent sample t-test was used for antibody type, sex, vital status, myeloablative conditioning regimen, and number of grade ≥3 toxicities. Pearson correlation for association was used for age and number of grade ≥3 toxicities. P values <.05 were considered statistically significant. All statistical analysis was performed using SPSS v.25.0 (IBM, USA).

### Literature Review

A literature search was conducted in PubMed/MEDLINE (January 1980–March 2020) to identify reports addressing toxicity of ch14.18 antibody-based immunotherapy in children treated for neuroblastoma. The search strategy and selection criteria can be found in **Supplementary Table 1**. To identify rare complications, case reports were included in this non-systematic review.

## Results

### Literature Review

To evaluate the toxicity associated with ch14.18 antibody-based immunotherapy in patients with neuroblastoma, we performed a review of the existing literature and identified six studies reporting grade ≥3 toxicities (2, 8, 9, 16–18). The most prevalent reported toxicities in these studies are listed in **Table 1**. Cross-study comparisons should be made with caution, since differences exists between these studies in antibody origin/manufacturer, concomitant cytokines administered, infusion times, and toxicity criteria used.

**Table 1:** Literature overview of reported non-hematological immunotherapy-related grade ≥3 toxicities.

Study - Year	Yu - 2010	Marachelian – 2016	Mody – 2017	Ladenstein - 2018	Mueller - 2018	Ozkaynak - 2018
Patients	n = 137	n = 28	n = 16^a^	n = 406	n = 53	n = 105
**Immunotherapy composition**						
Antibody	Ch14.18/NCI	Ch14.18/UTC^b^Ch14.18/NCI^b^	Dinutuximab	Dinutuximab beta	Dinutuximab beta^c^	Dinutuximab
Cytokines	IL-2 + GM-CSF	IL-2 + GM-CSF	GM-CSF	IL-2 (randomized)^d^	IL-2	IL-2 + GM-CSF
Other	Isotretinoin	Isotretinoin	Temozolomide/ Irinotecan	Isotretinoin	Isotretinoin	Isotretinoin
**Toxicity (%)**						
Pain	52	33 vs.29	44	16 vs.26^d^	38	22 – 41^e^
Fever	39	48 vs.44	25 (+ infection)	14 vs.40^d^	9	5 – 59^e^
Infection	39	n.r.	n.r.	25 vs.33^d^	n.r.	n.r.
CRI	13	n.r.	n.r.	n.r.	n.r.	n.r.
Hypotension	18	7 vs.11	13	4 vs.17^d^	2	4 – 17^e^
Hypersensitivity	25	n.r.	n.r.	10 vs.20^d^	2	2 – 10 ^e^ (AR)
Urticaria	13	n.r.	n.r.	5 vs.10	8	n.r.
CLS	23	n.r.	0	4 vs.15^d^	13	0 – 4^e^
Hypokalemia	35	26 vs.26	38	n.r.	n.r.	n.r.
Hyponatremia	23	19 vs.19	19	n.r.	n.r.	n.r.
Increased ALT	23	15 vs.4	6	17 vs.23 ^d^ (+AST)	n.r.	n.r.
Hypoxia	13	4 vs.11	25	n.r.	6	n.r.
Neurotoxicity						
Central	4	n.r.	n.r.	1.6 vs.5,8^d,f^	n.r.	n.r.
Peripheral	n.r.	n.r.	6	0.5 vs.3.1^d,g^	2	n.r.
Grade 5 toxicity (%)	1 (n = 1)	0	0	1 (n = 2)	0	1 (n = 1)
Antibody dose reductions (%)	n.r.	n.r.	38 (6/16)	n.r.	n.r.	43 (45/104)
Discontinuation IT due to toxicity (%)	15 (16/107)	7 (2/28)	13 (2/16)	5 (9/183) vs.16 (31/188)	n.r.	8 (8/104)

CRI, catheter-related infection; CLS, capillary leak syndrome; ALT, Alanine transaminase; AST, Aspartate transaminase; AR, allergic reaction; IT, immunotherapy; n.r., not reported. ^a^Maintenance + relapsed/refractory patients. ^b^Randomized crossover study comparing ch14.18-UTC (United Therapeutics Corporation) with ch14.18-NCI (National Cancer Institute). ^c^24 h continuous infusion. ^d^Comparison of immunotherapy with and without IL-2. ^e^Reported as range for courses 1–5. ^f^Disorientation/hallucinations, seizures, posterior reversible encephalopathy syndrome, toxic demyelinating encephalopathy + coma. ^g^Paresthesia, motor deficits, tetraparesis.

Overall, the most common grade ≥3 toxicity observed is pain with incidence rates ranging from 16% (without IL-2) to 62% (with IL-2) (8, 9). Pain occurred most frequently during the first immunotherapy course and at lower rates in subsequent courses (2, 8, 9, 16, 18). Grade ≥3 infections are reported in a range of 25%–39% (2, 8, 17), and fever in a range of 9%–67% (9, 18), with more grade ≥3 fever occurring in courses containing IL-2 (9). Seventy-seven percent more infections and 86% more catheter-related infections were seen in the immunotherapy group in comparison with standard isotretinoin therapy (2).

In three studies, grade 5 toxicity (death) was reported with an incidence rate of 1% (2, 8, 9). Causes of death were capillary leak syndrome (2×) (2, 8), in one case after an IL-2 overdose (medication error) (2), sudden cardiac arrest (9), and acute respiratory distress syndrome in the context of an infection (8).

The number of patients that permanently discontinued immunotherapy due to toxicity ranges from 5% to 16% (2, 8, 9, 16, 17). Half of the treatment discontinuations were caused by allergic reactions (9, 16, 17).

Several case reports and series have described rare side effects of ch14.18 antibody-based immunotherapy. Central neurotoxicity ranges in severity from disorientation and confusion (2, 8) to disabling cases of myelitis (19) and encephalopathy (2, 8, 20, 21), which warrant corticosteroid treatment and immediate discontinuation of immunotherapy. Full recovery is most likely to occur, although exceptions have been described (8, 21).

Ocular complications as mydriasis and accommodation deficits are frequently encountered and seldomly severe (8, 9, 22). Ocular symptoms are completely reversible in most patients and discontinuation of immunotherapy does not seem to be warranted (22).

Unusual and severe gastrointestinal complications have been associated with ch14.18 antibody-based immunotherapy in the form of necrotizing enterocolitis (23) and small bowel pneumatosis and ischemia (24).

### Patient Characteristics

Between 2016 and 2019, a cohort of twenty-six consecutive high-risk neuroblastoma patients (11 girls and 15 boys) were treated with dinutuximab-based immunotherapy and were included in this study. Patient characteristics are summarized in **Table 2**. The median age at diagnosis was 3.5 years (range 4 months–18 years). Most patients were INRG stage M and ≥ 12 months of age at diagnosis (n=22), while some were children with INRG stage L2 with *MYCN* amplification (n=4). The median time between diagnosis and the start of immunotherapy was 10 months (range 8–23 months). Five patients received dinutuximab, 20 patients received dinutuximab beta, and one patient received both antibodies.

**Table 2:** Patient characteristics of high-risk neuroblastoma cohort.

Patient characteristics			n = 26 No.	(%)
**Sex**				
	Female		11	42
	Male		15	58
**Age at diagnosis** (months)				
	Median		41.5	
	Range		4–224	
**Age at start immunotherapy** (months)				
	Median		55	
	Range		16–240	
**INRG stage at diagnosis**^a^				
	Stage L2		4	15
	Stage M		22	85
**Location primary tumor**				
	Adrenal		19	73
	Sympathetic side chain		7	27
***MYCN* status**				
	Amplified		11	42
	Single copy		15	58
**Treatment – Induction**				
	Standard induction chemotherapy + Surgery		18	69
		+ additional (chemo)therapy	8	12
**Treatment - Consolidation – Myeloablative conditioning regimen**				
	Carboplatin/Etoposide/Melphalan (CEM)		9	35
	Busulfan/Melphalan (BuMel)		17	65
**Treatment – Maintenance - Anti-GD2 antibody**				
	Dinutuximab		5	19
	Dinutuximab beta		20	77
	Both antibodies		1	4
**Disease status at start immunotherapy**^b^				
	Complete response (CR)		14	54
	Partial response (PR)		12	46
**Vital status at end of follow-up**				
	Alive		20	77
	Dead		6	23
**Follow-up** (End immunotherapy – Last control; months)				
	Median		22	
	Range		9 – 39	

^a^As defined by International Neuroblastoma Risk Group (11). ^b^As defined by International Neuroblastoma Response Criteria (25).

### Toxicities

Twenty-three patients completed all six courses of immunotherapy. In two patients, immunotherapy was discontinued after two courses. One patient developed unacceptable toxicities: grade 4 catheter-related infection (CRI) in combination with bilateral mydriasis and severe peripheral sensory and motor neuropathy which improved over time but did not resolve completely. In another patient, immunotherapy was discontinued because of disease progression. In a third patient, isotretinoin was permanently discontinued during course 5 due to liver toxicity (grade 4 elevated AST and ALT).

**Figure 1:**
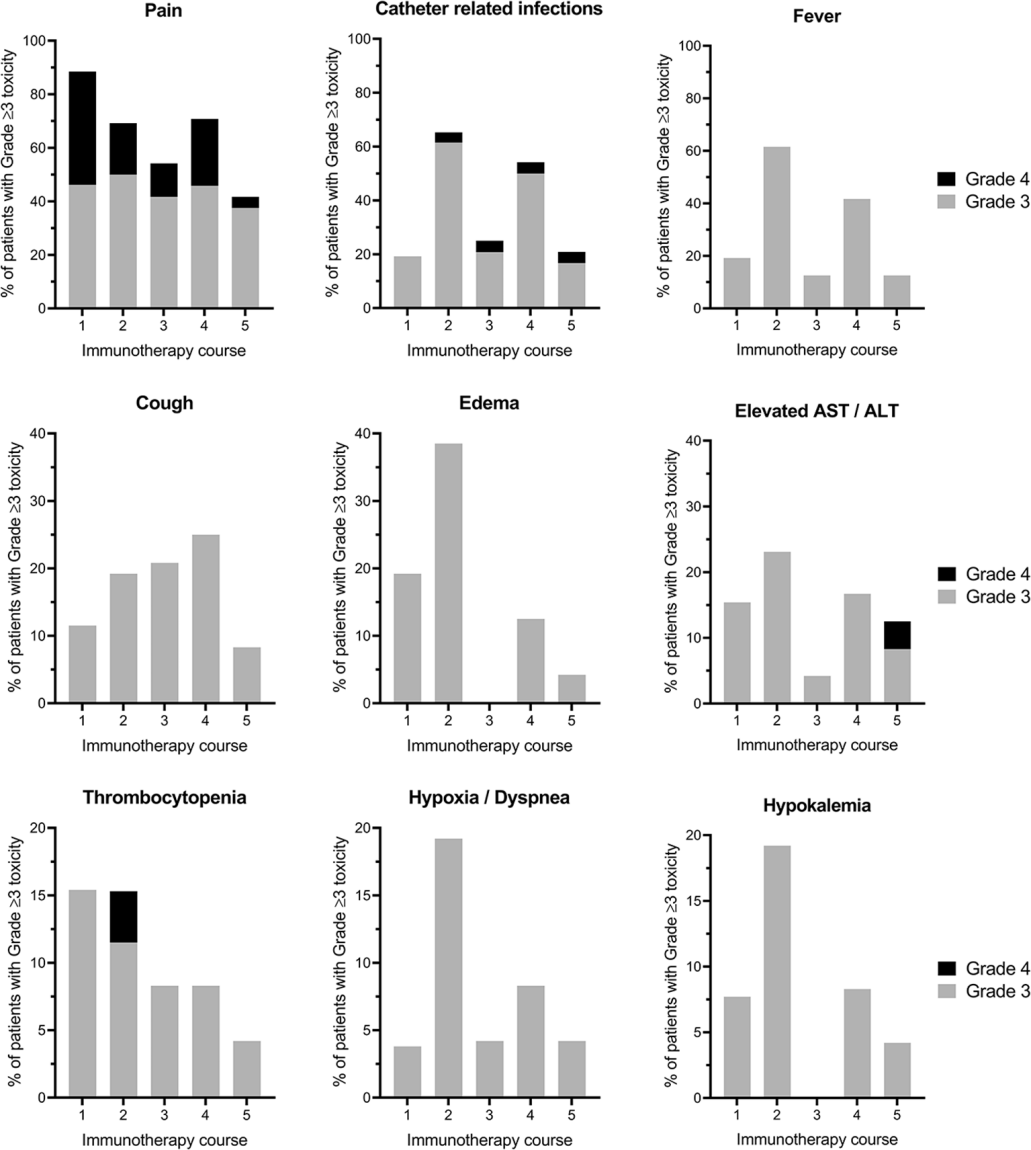
The most prevalent immunotherapy-related grade ≥3 toxicities per course. Proportion of patients experiencing grade ≥3 toxicities per cycle (1–5) of immunotherapy are displayed. The highest grade of toxicity per patient per course is shown. Please note the different scales of the Y-axis. AST, Aspartate transaminase; ALT, Alanine transaminase.

In total, 310 grade ≥3 toxicities were recorded during 124 immunotherapy courses (courses 1–5). Grade ≥3 toxicities were not evenly distributed among the five courses; with most toxicities reported in courses 2 and 4 involving IL-2 (20; 36; 12; 23; 10% for courses 1–5, respectively). All 26 patients experienced at least one grade ≥3 toxicity. Thirty-three grade 4 toxicities in 19/26 patients and no grade 5 toxicities (death) were seen. In **Figure 1**, the most common grade ≥3 toxicities for courses 1–5 are depicted, while all toxicities encountered during the immunotherapy courses are listed in **Supplementary Table 2**. Here only the highest grade per course per patient is given. Pain was the most common grade ≥3 toxicity observed in 96% (25/26) of patients at some point during immunotherapy (courses 1–5). Sixty-five percent (17/26) of patients suffered from disabling, grade 4 pain. Esketamine and clonidine were used in 88% (23/26) and 50% (13/26) of patients, respectively, due to inadequate pain control. Grade ≥3 pain was most frequent during immunotherapy course 1, occurring in 88% of patients. During course 5 the proportion of patients with grade ≥3 pain decreased to 42% (p=.003).

The second and third most common grade ≥3 toxicity were CRIs in 19%, 65%, 25%, 54%, 21%, and fever in 19%, 62%, 13%, 42%, 13% of patients in courses 1–5, respectively. Although both toxicities occurred more frequently in immunotherapy courses that contained IL-2 (courses 2 and 4) as compared with courses that contained GM-CSF (courses 1, 3, and 5), a statistically significant difference between IL-2 and GM-CSF courses was only found for catheter-related infections (p=.039), and not for fever (p=.057).

**Figure 2:**
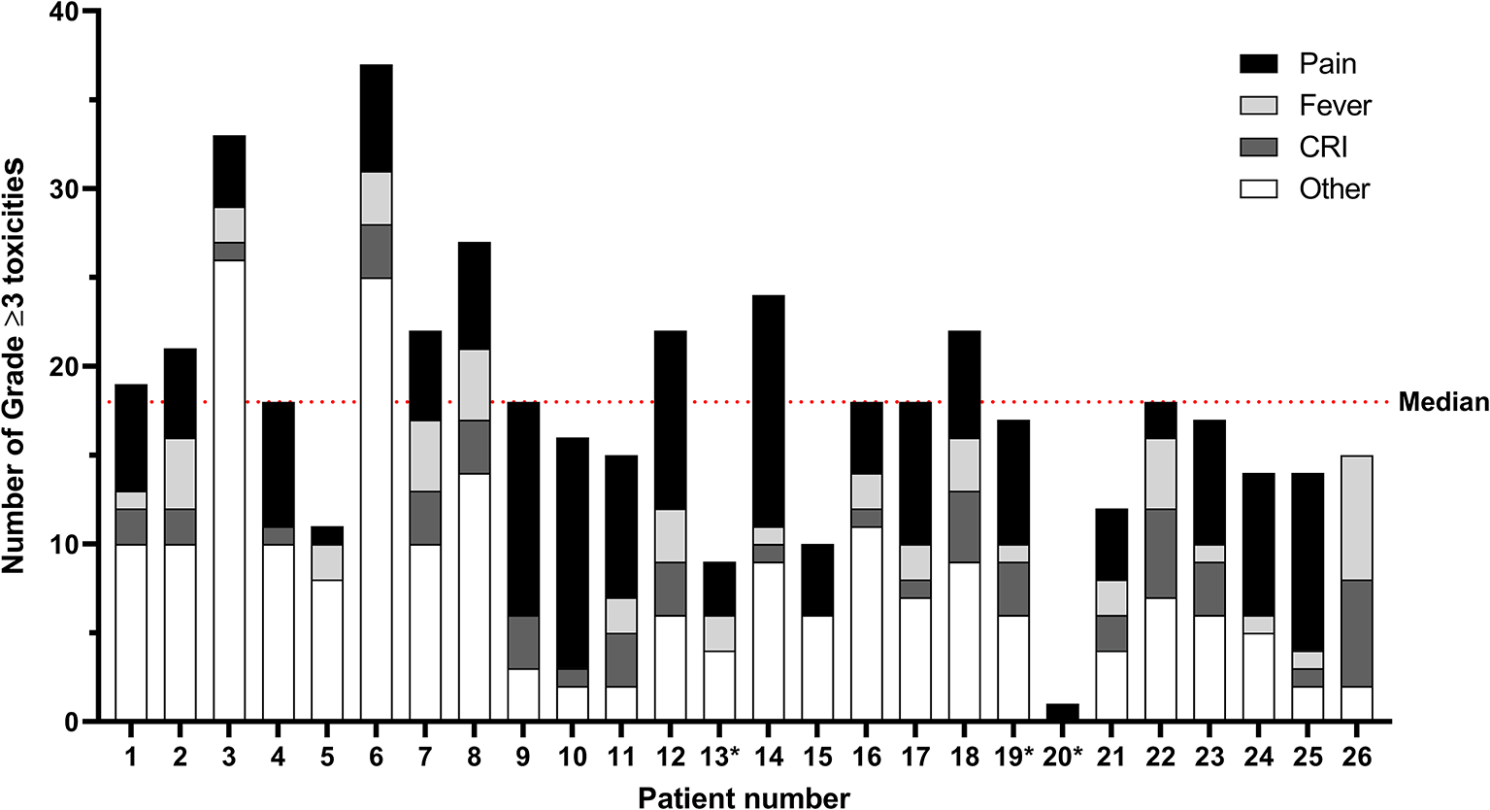
Immunotherapy-related grade ≥ 3 toxicities per patient for courses 1–5. Toxicities may be documented multiple times per course, with a maximum of once per day. The median number of grade ≥3 toxicities per patient is 18 (range 10–37) for the 23 patients who completed all six courses of immunotherapy. The category “Other” comprises toxicities in alphabetical order from the CTCAE categories Allergy; Blood/bone marrow; Constitutional, Cardiac; Gastrointestinal, Infection; Lymphatics; Metabolic/laboratory; Neurology; Ocular/visual; Pulmonary; and Vascular. All individual toxicities encountered are listed in **Supplementary Table 2**. *Patients 13, 19, and 20 did not complete all cycles of immunotherapy. CRI, catheter-related infections.

In **Figure 2** and **Supplementary Table 3**, all grade ≥3 toxicities per patient are shown for courses 1–5. Here toxicities may be documented multiple times per course, with a maximum of once per day. In total, 441 grade ≥3 toxicities were recorded for the 23 patients that completed all six courses of immunotherapy, with a median of 18 (range 10–37) grade ≥3 toxicities per patient. No statistically significant difference in the number of grade ≥3 toxicities per patient were noted between the patients that received dinutuximab or dinutuximab beta (p=.754). The same holds true for sex (p=.275), myeloablative conditioning regimen (p=.708), and vital status at the end of follow-up (p=.948). Age at diagnosis (p=.908) and at the start of immunotherapy (p=.925) were not significantly correlated with the number of grade ≥3 toxicities per patient.

### Fever Management

All 26 patients experienced fever during immunotherapy; 81% of patients suffered from fever >40.0°C (grade 3). In 124 courses of immunotherapy (courses 1–5), 341 instances of fever were recorded (**Table 3**). Sixty-three percent of fever episodes were recorded in the immunotherapy courses with IL-2 (course 2: 34% and course 4: 29%). During these episodes, 274 blood cultures were taken from which 52 CRIs were identified in 81% of patients. Four of these episodes were life-threatening (grade 4) and led to intensive care admission followed by full recovery. All 26 patients received immunotherapy through a Hickman central venous access device (CVAD). Twelve patients (46%) had a Hickman CVAD implanted shortly before the start of immunotherapy, the other patients (54%) received a Hickman CVAD earlier in their treatment history (i.e., induction or consolidation phase). Surgical removal of the CVAD was performed in 54% (28/52) of CRIs. Ten patients (38%) did not require a CVAD removal during immunotherapy.

**Table 3:** Fever instances, blood cultures, and catheter-related infections.

		Course 1 (n = 26)		Course 2 (n = 26)		Course 3 (n = 24)		Course 4 (n = 24)		Course 5 (n = 24)		Total
		No.	No. patients	No.	No. patients	No.	No. patients	No.	No. patients	No.	No. patients	No.
**Fever instances**		56	25	115	26	40	20	99	24	31	18	341
**Grade (in %)**	**1** **2** **3**	41 48 11		23 55 23		73 15 13		28 60 12		68 23 10		
**Blood cultures**		48	24	99	26	42	17	68	22	17	12	274
**Positive BC**		15	11	42	22	18	11	34	16	7	7	116
**CRI**		6	5	20	17	7	6	14	13	5	5	52
**CVAD out**		3		10		5		7		3		28
**PICU admission**		0		1		1		1		1		4
**CRI pathogens**												
Staphylococcus sp.		1		7		1		5		1		15
Streptococcal sp.		2		5		1		3		2		13
Gram-negative sp.		2		2		1		5		2		12
Mixed Gram pos + neg sp.		0		1		1		1		0		3
Less/non-pathogenic sp.		1		5		3		0		0		9

The maximum body temperature per day was used to assess fever. Days with one recorded body temperature of ≥38.0°C were counted as fever instances. Grades 1, 2, 3 indicate the percentual distribution between Fever grades 1, 2, and 3, respectively. BC, blood culture; CRI, catheter-related infections; CVL, central venous access device; PICU, pediatric intensive care unit; pos, positive; neg, negative; sp., species.

Staphylococcus species were identified in 29% (15/52) of CRIs, in 14/15 blood cultures a Staphylococcus aureus was isolated. In 29% (15/52) of CRIs, Gram-negative pathogens were detected. **Supplementary Table 4** lists the identified bacteria isolated in all CRIs.

### Dose Modifications

To reduce the toxicity burden of immunotherapy, the infusion rate of dinutuximab may be decreased. In case of more severe toxicity, temporary interruption or permanent discontinuation of infusion may be necessary. In our cohort, the dinutuximab dose was modified in 81% (21/26) of patients (**Table 4**). Forty-two percent (11/26) of patients did not receive 100% of the intended dinutuximab dose at some point during courses 1–5. In courses 1 and 3, all patients received 100% of the intended dinutuximab dose. In course 2, 19% (5/26) did not receive the planned dinutuximab dose. Here, two patients, both suffering from a CRI, only received <50%. In course 4, 21% (5/24) received ≥50 to <100% of cumulative intended dinutuximab dose. Lastly, in course 5, 8% (2/24) did not receive the planned dinutuximab dose. Here, one patient received <50% of the dinutuximab dose after therapy discontinuation due to severe coughing.

**Table 4:** Immunotherapy-related toxicities leading to dose modifications of dinutuximab.

Course(n=)	100% of cumulative intended dose of dinutuximab administered			≥ 50 to <100% of cumulative intended dose of dinutuximab administered			<50% of cumulative intended dose of dinutuximab administered		
	No. patients	No. patients with dose modifications	Indication for dose modification	No. patients	No. patients with dose modifications	Indication for dose modification	No. patients	No. patients with dose modifications	Indication for dose modification
**Course 1(26)**	26 (100%)	8/26	1× AR 3× Cough 2× Pain 1× Pain + Cough 1× Pain + Fever	0 (0%)			0 (0%)		
**Course 2(26)**	21 (81%)	3/21	3× Cough	3 (12%)	3/3	1× AR + Fever 1× Cough 1× Hypertension	2 (8%)	2/2	2× CRI
**Course 3(24)**	24 (100%)	6/24	6× Cough	0 (0%)			0 (0%)		
**Course 4(24)**	19 (79%)	7/19	5× Cough 1× Pain + Cough 1× Hypertension	5 (21%)	5/5	1× AR 2× CRI 1× Fever 1× Hypoxia	0 (0%)		
**Course 5(24)**	22 (92%)	4/21	1× AR 1× Cough 1× Fever 1× Pain	1 (4%)	1/1	1× CRI	1 (4%)	1/1	1× Cough

AR, allergic reaction; CRI, catheter-related infection.

Although more patients did not receive 100% of the intended dinutuximab dose in the courses containing IL-2 (n=9) as compared with courses containing GM-CSF (n=2), this difference was not significant (p=.065). Grade ≥3 pain, the most common toxicity overall, led to dose modifications in 5 patients. All five patients, however, received 100% of cumulative intended course dose of dinutuximab.

The treatment of patients with IL-2 had to be modified due to treatment-related toxicities in 54 and 48% of patients in courses 2 and 4, respectively (**Supplementary Table 5**). The most prevalent toxicities preceding IL-2 dose modifications were coughing, CRIs, AST/ALT abnormalities, and fever.

### Disease Outcome

At the last follow-up, six patients (23%) had died of disease after the start of immunotherapy. One patient, with a complete response before the start of immunotherapy, suffered from cerebral metastases and only completed two courses of immunotherapy. The other five patients had a relapse after immunotherapy completion. In four of these patients, skeletal relapses were detected at the response assessment after immunotherapy course 6. In the fifth patient, a mediastinal soft tissue relapse was discovered 8 months after the completion of immunotherapy.

## Discussion

Treatment-related toxicity during immunotherapy with dinutuximab, cytokines IL-2 and GM-CSF, and isotretinoin after induction and consolidation according to the DCOG NBL 2009 treatment protocol is substantial. All 26 analyzed patients suffered from grade ≥3 toxicities, 73% suffered from grade 4 toxicities and no grade 5 toxicities (death) were seen. Pain, fever, coughing, edema, and liver enzyme abnormalities were among the most common toxicities observed. These results are in line with earlier reports in which comparable immunotherapy regimens were used (2, 9, 16). A large interpatient variability in grade ≥3 toxicity burden was observed, possibly related to the pharmacokinetic variability of dinutuximab in disposition and clearance in children (26, 27). Generally, immunotherapy-related toxicities were transient and resolved with the discontinuation of antibody and/or cytokine infusion, or with appropriate supportive care (pain/fever management). However, 12% of patients (3/26) could not complete all immunotherapy courses due to toxicity, and in one of these patients the peripheral neuropathy did not resolve completely. Peripheral neurotoxicity is a rare, but severe side effect of immunotherapy with a reported prevalence between 2 and 6% (8, 17, 18).

Pain was most severe during the first course and significantly improved during subsequent courses. In our results, 88% of patients experienced grade ≥3 pain in course 1, whereas 41% experienced pain in course 5. Comparable reductions in the proportion of patients experiencing grade ≥3 pain between courses 1 and 5 were seen in the studies of Yu et al. (37%–14%) and Ozkaynak et al. (41%–24%) (2, 9). Decreasing pain scores and intravenous morphine usage in time within and between immunotherapy courses were also observed by Mueller et al. in a study evaluating long-term dinutuximab infusion (LTI, continuous 10-day antibody infusion) (18). Accelerated antibody clearance after repeated administration of dinutuximab may explain the observed decreasing proportion of patients experiencing grade ≥3 pain over the courses (27). However, this explanation of accelerated antibody clearance was contested by another, more recent study (26). The improved pain tolerance in subsequent cycles could also be explained by the individualization of pain management. The individual and initial pain response would guide the subsequent pain management, making it more effective in subsequent courses.

Eighty-one percent of patients (21/26) suffered from catheter-related infections during anti-GD2 immunotherapy. This percentage is remarkably higher than reported previously (2). Most catheter-related infections (65%) were recorded in the immunotherapy courses containing IL-2 (courses 2 and 4). Previous studies have shown an increase in bacteremia and catheter-related infections in cancer patients receiving IL-2 (28–32). Staphylococcus aureus was cultured in 27% (14/52) of catheter-related infections in our study. This prevalence is strikingly higher than the 5.2% of S. aureus cultured during central line-associated bloodstream infection (CLABSI) episodes in a report on CVAD-related complications in pediatric oncology patients from colleagues at our institution (33). In unpublished data by Van den Bosch et al. on CVAD-related complications in neuroblastoma patients, significantly more S. aureus-CLABSIs and CLABSIs overall were observed in neuroblastoma patients receiving anti-GD2 immunotherapy. Strategies to prevent catheter-related infections have been studied, including the prophylactic use of antibiotics (34, 35) and the use of antibiotic-coated catheters (36). To our knowledge, no studies have examined the benefit of these strategies in this patient population.

The courses containing dinutuximab with IL-2 (courses 2 and 4) were associated with more toxicity than the courses with GM-CSF (courses 1, 3, 5), a result also encountered in other studies (2, 9). Moreover, 19% and 21% of patients did not receive the intended dose of dinutuximab due to toxicity in courses 2 and 4, respectively. In contrast, in the other three courses only in course 5 did 8% of patients not receive the complete dinutuximab dose. In one study, IL-2 was thought to be the causative agent in the majority of fever instances without documented infection (6). In another study by Ladenstein et al, patients were randomly assigned to receive either dinutuximab beta plus IL-2 or dinutuximab beta alone (8). No evidence was found that addition of IL-2 improved outcome. Furthermore, dinutuximab beta plus IL-2 was associated with greater toxicity, more dose modifications and less treatment completion than dinutuximab beta alone, leading the authors to conclude that dinutuximab immunotherapy without IL-2 should be considered standard of care.

The major limitation of our study is the small size of the cohort, making detection of rare complications of treatment less probable. Furthermore, early patients were treated with dinutuximab, while after EMA approval in May 2017 patients were treated with dinutuximab beta. We, however, found no difference in number of grade ≥3 toxicities per patient between the two antibodies and evidence exists that both antibodies have comparable toxicity profiles (37). Lastly, the retrospective nature of our study is a potential source of bias. Although, immunotherapy was newly introduced in our center and all healthcare providers involved were instructed in accurate toxicity recordkeeping, information and selection bias cannot be ruled out completely.

The strength of our study is that all toxicities were uniformly collected, categorized, and graded by a small group with extensive experience in toxicity reporting of cancer treatment in children. We believe that this design in combination with retrospective collection of toxicities from patients’ medical files led to more sensitive toxicity collection, and therefore to higher prevalences of toxicities than previously reported (2, 8, 9, 16–18).

We conclude in this single center experience of immunotherapy with dinutuximab, cytokines IL-2 and GM-CSF, and isotretinoin after induction and consolidation according to the DCOG NBL 2009 treatment protocol, that immunotherapy-related toxicity is substantial, but manageable. Future studies are warranted to optimize the scheduling, anti-GD2 antibody (38), and additive cytokines of immunotherapy with anti-GD2 monoclonal antibodies in high-risk neuroblastoma.

## Data Availability Statement

The raw data supporting the conclusions of this article will be made available by the authors, without undue reservation.

## Ethics Statement

The studies involving human participants were reviewed and approved by the Medical Research Ethics Committee Utrecht (info@metcutrecht.nl).

## Author Contributions

TB, RL, LA, MM, TW, MD, NE, KK, MN, MG, and GT contributed to the conception and design of the study. TB, RL, and GT organized the database. TB and RL collected the data. TB performed the statistical analysis. TB, RL, and GT wrote the first draft of the manuscript. LA, MM, and TW wrote sections of the manuscript. All authors contributed to the article and approved the submitted version.

## Conflict of Interest

The authors declare that the research was conducted in the absence of any commercial or financial relationships that could be construed as a potential conflict of interest.

## Abbreviations

GD2, Disialoganglioside 2; IL-2, Interleukin-2; GM-CSF, Granulocyte-macrophage colony-stimulating factor; ADCC, Antibody-dependent cellular cytotoxicity; COG, Children’s Oncology Group; DCOG, Dutch Childhood Oncology Group; INRG, International Neuroblastoma Risk Group; GPOH, Gesellschaft für Pädiatrische Onkologie und Hämatologie; IV, intravenous; CTCAE, Common Terminology Criteria for Adverse Events; WB-FPRS, Wong–Baker Faces Pain Rating Scale; VAS, Visual analogue scale; CRI, Catheter-related infection; PICU, Pediatric intensive care unit; SPSS, Statistical Package for the Social Sciences; ALT, Alanine transaminase; AST, Aspartate transaminase; LTI, long-term infusion; EMA, European Medicines Agency; CVAD, central venous access device; CLABSI, central line-associated bloodstream infection.
