# Supplementary material for: Treatment-Related Toxicities During Anti-GD2 Immunotherapy in High-Risk Neuroblastoma Patients
Source: Front Oncol. 2021 Feb 17;10:601076. doi: 10.3389/fonc.2020.601076 (PMC7925836; doi:10.3389/fonc.2020.601076)
Supplement: Supplementary file 1 [file Image_1.pdf]

Supplementary Figure 1. Overview of immunotherapy courses 1 – 6

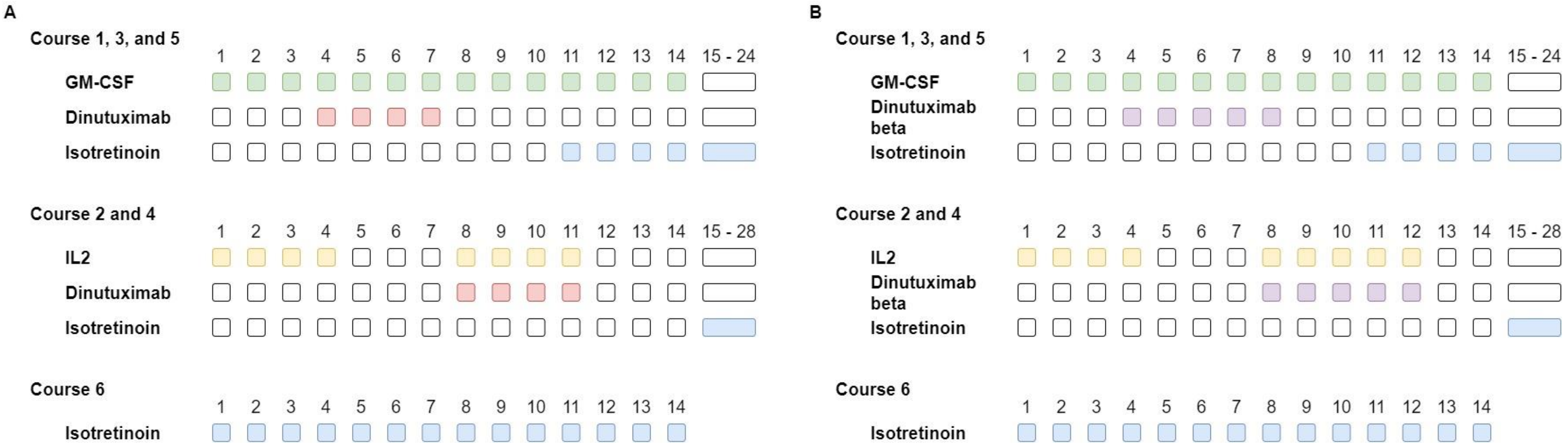

**A = Dinutuximab immunotherapy treatment schema**	**B = Dinutuximab beta immunotherapy treatment schema**
**Dinutuximab**<br>ch14.18/SP2/0 (Murine myeloma cells)<br>Dosing regimen: 17.5 mg/m^2 given by 10 hour infusion (max 20 hours) on 4 consecutive days<br>Cumulative dose: 70 mg/m^2 per cycle	**Dinutuximab beta**<br>ch14.18/CHO (Chinese hamster ovary cells)<br>Dosing regimen: 20 mg/m^2 given by 8 hour infusion (max 16 hours) on 5 consecutive days<br>Cumulative dose: 100 mg/m^2 per cycle
**Interleukin-2 (IL-2)**<br>3.0 x 10^6 IU/m^2/day on 4 consecutive days by IV continuous infusion in week 1<br>4.5 x 10^6 IU/m^2/day on 4 consecutive days by IV continuous infusion in week 2	**Interleukin-2 (IL-2)**<br>3.0 x 10^6 IU/m^2/day on 4 consecutive days by IV continuous infusion in week 1<br>4.5 x 10^6 IU/m^2/day on 5 consecutive days by IV continuous infusion in week 2
**GM-CSF (Sargramostim)**<br>250 µg/m^2/day on 14 consecutive days (s.c.)	**GM-CSF (Sargramostim)**<br>250 µg/m^2/day on 14 consecutive days (s.c.)
**Isotretinoin (13-cis-RA)**<br>160 mg/m^2/day on 14 consecutive days	**Isotretinoin (13-cis-RA)**<br>160 mg/m^2/day on 14 consecutive days

IV = intravenous; s.c. = subcutaneous.
