# Supplementary material for: Treatment-Related Toxicities During Anti-GD2 Immunotherapy in High-Risk Neuroblastoma Patients
Source: Front Oncol. 2021 Feb 17;10:601076. doi: 10.3389/fonc.2020.601076 (PMC7925836; doi:10.3389/fonc.2020.601076)
Supplement: Supplementary file 2 [file Table_1.pdf]

Supplementary Table 1. Search strategy and inclusion criteria

Search strategy for MEDLINE (PubMed)

1. For ch14.18 antibody we used the following MeSH headings and text words:

"antibodies, monoclonal/therapeutic use"[mh] OR "gangliosides/therapeutic use"[mh] OR immunotherapy/methods[mh] OR anti gd2[tiab] OR ch14 18 OR ganglioside* OR dinutuximab OR unituxin

2. For Neuroblastoma we used the following MeSH headings and text words:

neuroblastoma OR neuroblastomas OR neuroblast* OR ganglioneuroblastoma OR ganglioneuroblastomas OR ganglioneuroblast* OR neuroepitheliom*

Final search 1 and 2

[*=zero or more characters; mh=MeSH term; tiab=title or abstract]

The reference lists of all relevant articles were screened for additional references not registered in PubMed/MEDLINE.

Criteria for considering studies for this review

Types of studies: Any study design accepted, including case reports. Conference abstracts will not be included. (Systematic) Reviews containing no primary data will not be included.

Types of participants: Patients with histologically proven neuroblastoma, irrespective of prior therapy.

Types of intervention: Treatment with ch14.18 (dinutuximab) antibody-based immunotherapy, with or without the addition of immunostimulatory cytokines [interleukin-2 (IL-2) and granulocyte-macrophage colony-stimulating factor (GM-CSF)]. Studies in which patients were treated with 3F8 antibody-based immunotherapy were **not** included in this review.

Types of outcome measures: Acute toxicity, hematological and non-hematological adverse events (AEs) and late effects, as defined in including studies.
