# Supplementary material for: Treatment-Related Toxicities During Anti-GD2 Immunotherapy in High-Risk Neuroblastoma Patients
Source: Front Oncol. 2021 Feb 17;10:601076. doi: 10.3389/fonc.2020.601076 (PMC7925836; doi:10.3389/fonc.2020.601076)
Supplement: Supplementary file 3 [file Table_2.pdf]

Supplementary Table 2. Overview of all grade ≥3 toxicities per course
gr = grade; - = no cases / not reported; CRI = catheter related infection; Sx = symptoms; AST = Aspartate transaminase; ALT = Alanine transaminase; GGT = Gamma-glutamyltransferase.
* Infection; Other comprises of: 1) Skin infection, Herpes Zoster; 2) Gastroenteritis, Clostridium; 3) Gastroenteritis, Salmonella; 4) Biliary tree / Cholangitis, unknown pathogen; 5) Upper air way, unknown pathogen; 6) Oral cavity, fungal. ** Classification and grading according to CTCAE version 5.0.

**Category**	**Course 1 (n = 26)**		**Course 2 (n = 26)**		**Course 3 (n = 24)**		**Course 4 (n = 24)**		**Course 5 (n = 24)**		**Total**
Toxicity	gr 3	gr 4	gr 3	gr 4	gr 3	gr 4	gr 3	gr 4	gr 3	gr 4	
**Pain**											
Pain	12	11	13	5	10	3	11	6	9	1	81
**Infection**											
CRI	5	-	16	1	5	1	12	1	4	1	46
Infection; other*	1	-	2	-	1	-	2	-	-	-	6
**Constitutional Sx**											
Fever	5	-	16	-	3	-	10	-	3	-	37
Fatigue	-	-	1	-	-	-	-	-	-	-	1
**Pulmonary**											
Cough	3	-	5	-	5	-	6	-	2	-	21
Hypoxia / Dyspnea	1	-	5	-	1	-	2	-	1	-	10
**Lymphatics**											
Edema	5	-	10	-	-	-	3	-	1	-	19
**Metabolic/Laboratory**											
AST and/or ALT	4	-	6	-	1	-	4	-	2	1	18
Hypokalemia	2	-	5	-	-	-	2	-	1	-	10
GGT	2	-	3	-	2	-	1	-	1	-	9
Hypophosphatemia	-	-	1	-	-	-	-	-	-	-	1
Bilirubin	-	-	1	-	-	-	-	-	-	-	1
Creatinine	-	-	-	-	-	-	1	-	-	-	1
**Blood/Bone marrow**											
Thrombocytopenia	4	-	3	1	2	-	2	-	1	-	13
Hemoglobin	-	-	1	-	1	-	1	-	-	-	3
Lymphopenia and/or Neutrophils	1	-	2	-	-	-	-	-	-	-	3
**Cardiac**											
Hypotension	2	-	4	-	-	-	2	-	-	-	8
Hypertension	-	-	2	-	1	-	1	-	-	-	4
**Allergy**											
Allergic reaction	-	-	4	-	-	-	3	-	1	-	8
**Gastrointestinal**											
Diarrhea	2	-	-	-	1	-	-	-	-	-	3
Nausea	-	-	-	-	-	-	-	-	1	-	1
**Neurology**											
Confusion	1	-	-	-	-	-	-	-	-	-	1
Neuropathy motor / sensory	-	-	1	-	-	-	-	-	-	-	1
**Ocular/Visual**											
Blurred vision	-	-	1	-	-	-	-	-	-	-	1
Mydriasis	-	-	1	-	-	-	-	-	-	-	1
**Vascular****											
Capillary leak syndrome	-	-	1	1	-	-	-	-	-	-	2
**Total**	**50**	**11**	**104**	**8**	**33**	**4**	**63**	**7**	**27**	**3**	**310**
