# Supplementary material for: Treatment-Related Toxicities During Anti-GD2 Immunotherapy in High-Risk Neuroblastoma Patients
Source: Front Oncol. 2021 Feb 17;10:601076. doi: 10.3389/fonc.2020.601076 (PMC7925836; doi:10.3389/fonc.2020.601076)
Supplement: Supplementary file 4 [file Table_3.pdf]

Supplementary Table 3: Grade ≥3 toxicities per patient for immunotherapy courses 1 – 5

F = female; M = male; D = dinutuximab; DB = dinutuximab beta; Y = Yes; N = No; Mo = months; d = days; MAT = myeloablative therapy; IT = immunotherapy; CRI = catheter related infection; gr = grade; BuMel = Busulfan, Melphalan; CEM = Carboplatin, Etoposide, Melphalan; N/A = not applicable. The category "Other" comprises toxicities in alphabetical order from the CTCAE categories Allergy; Blood/bone marrow; Constitutional, Cardiac; Gastrointestinal, Infection; Lymphatics; Metabolic/laboratory; Neurology; Ocular/visual; Pulmonary; and Vascular. * Patient received dinutuximab in course 1 and 2, and dinutuximab beta in course 3-5.

Patient number	Sex	Antibody	Age diagnosis (mo)	Vital status	MAT	IT completed	Interval start IT – end IT (d)	Interval diagnosis – start IT (d)	Fever (gr ≥ 3)	CRI (gr ≥ 3)	Pain (gr ≥ 3)	Other (gr ≥ 3)	Total (gr ≥ 3)
**1**	F	D	78	dead	CEM	Y	178	351	1	2	6	10	**19**
**2**	F	D	59	alive	CEM	Y	178	322	4	2	5	10	**21**
**3**	F	D	19	alive	CEM	Y	178	367	2	1	4	26	**33**
**4**	M	D	224	dead	BuMel	Y	178	490	0	1	7	10	**18**
**5**	M	D	14	alive	CEM	Y	178	288	2	0	1	8	**11**
**15**	M	D+DB*	7	alive	BuMel	Y	178	269	0	0	4	6	**10**
**6**	M	DB	41	alive	BuMel	Y	184	309	3	3	6	25	**37**
**7**	M	DB	21	dead	CEM	Y	205	312	4	3	5	10	**22**
**8**	F	DB	9	alive	CEM	Y	191	596	4	3	6	14	**27**
**9**	F	DB	133	alive	CEM	Y	177	715	0	3	12	3	**18**
**10**	M	DB	38	alive	CEM	Y	177	350	0	1	13	2	**16**
**11**	F	DB	4	alive	BuMel	Y	177	399	2	3	8	2	**15**
**12**	F	DB	42	alive	BuMel	Y	198	413	3	3	10	6	**22**
**13**	F	DB	21	alive	BuMel	N	N/A	315	2	0	3	4	**9**
**14**	M	DB	52	alive	BuMel	Y	177	324	1	1	13	9	**24**
**16**	F	DB	37	alive	BuMel	Y	184	511	2	1	4	11	**18**
**17**	M	DB	48	dead	BuMel	Y	177	309	2	1	8	7	**18**
**18**	F	DB	61	alive	BuMel	Y	191	312	3	4	6	9	**22**
**19**	M	DB	55	alive	BuMel	N	N/A	310	1	3	7	6	**17**
**20**	M	DB	29	dead	BuMel	N	N/A	254	0	0	1	0	**1**
**21**	M	DB	51	alive	CEM	Y	205	421	2	2	4	4	**12**
**22**	M	DB	56	dead	Bumel	Y	212	322	4	5	2	7	**18**
**23**	M	DB	47	alive	BuMel	Y	198	455	1	3	7	6	**17**
**24**	M	DB	27	alive	BuMel	Y	177	310	1	0	8	5	**14**
**25**	F	DB	75	alive	BuMel	Y	177	393	1	1	10	2	**14**
**26**	M	DB	9	alive	BuMel	Y	240	320	7	6	0	2	**15**
