# Supplementary material for: Treatment-Related Toxicities During Anti-GD2 Immunotherapy in High-Risk Neuroblastoma Patients
Source: Front Oncol. 2021 Feb 17;10:601076. doi: 10.3389/fonc.2020.601076 (PMC7925836; doi:10.3389/fonc.2020.601076)
Supplement: Supplementary file 5 [file Table_4.pdf]

Supplementary Table 4: Identified bacteria in catheter-related infections

Course 1	Course 2	Course 3	Course 4	Course 5
**Considered pathogenic (Gram pos)**<br>* Staph aureus<br><br>* Strept mitis (+ Staph epidermidis, Micrococcus luteus)<br>* Strept pneumoniae	**Considered pathogenic (Gram pos)**<br>* Staph aureus (4x)<br>* Staph aureus (+ Staph epidermidis)<br>* Staph aureus (+ Staph hominis)<br>* Staph aureus (+ Bacillus cereus)<br><br>* Strept bovis (+ Staph hominis, Micrococcus luteus)<br>* Strept mitis (+ Staph epidermidis)<br>* Strept mitis (+ Staph epidermidis, Staph hominis)<br>* Strept mitis, Strept salivarius (+ Staph epidermidis)<br>* Strept pyogenes	**Considered pathogenic (Gram pos)**<br>* Staph aureus<br><br>* Strept mitis (+ Staph epidermidis)	**Considered pathogenic (Gram pos)**<br>* Staph aureus<br>* Staph aureus (+ Staph epidermidis, Staph haemolyticus)<br>* Staph aureus (+ Staph epidermidis, Staph pettenkoferi, Enterococcus faecium)<br>* Staph aureus, Strept mitis (+ Staph hominis, Enterococcus faecalis)<br>* Staph haemolyticus (+ Staph epidermidis, Staph hominis)<br><br>* Strept mitis<br>* Strept mitis (+ Gemella haemolysans, Actinomyces oris, Rothia dentocariosa)<br>* Strept pneumoniae	**Considered pathogenic (Gram pos)**<br>* Staph aureus<br><br>* Strept mitis (Bacillus lichenformis, Abiotrophia defective)<br>* Strept salivarius
**Considered pathogenic (Gram neg)**<br>* Enterobacter cloacae (2x) *	**Considered pathogenic (Gram neg)**<br>* Enterobacter cloacae<br>* Klebsiella oxytoca	**Considered pathogenic (Gram neg)**<br>* Moraxella osloensis (+ Micrococcus luteus)	**Considered pathogenic (Gram neg)**<br>* Acinetobacter lwoffii (+Staph epidermidis)<br>* Enterobacter cloacae<br>* Klebsiella oxytoca, Klebsiella aerogenes, Acinetobacter species (+ Staph epidermidis)<br>* Moraxella catarrhalis<br>* Pantoa septica, Pseudomonas oryzihabitans (+ Bacillus cereus, Staph auricularis, Staph epidermidis)	**Considered pathogenic (Gram neg)**<br>* Klebsiella pneumoniae<br>* Serratia marcescens
	**Considered pathogenic (Gram pos/neg)**<br>* Strept pneumoniae, Moraxella catarrhalis	**Considered pathogenic (Gram pos/neg)**<br>* Strept mitis, Pantoea agglomerans (+ Micrococcus luteus)	**Considered pathogenic (Gram pos/neg)**<br>* Strept pneumoniae, Moraxella catarrhalis (+ Enterococcus faecalis)	
**Considered less/non-pathogenic**<br>* Staph epidermidis	**Considered less/non-pathogenic**<br>* Staph epidermidis (3x)<br>* Staph hominis<br>* Enterococcus faecalis, Staph epidermidis	**Considered less/non-pathogenic**<br>* Staph epidermidis (2x)<br>* Staph epidermidis, Staph hominis		

* = two Enterobacter cloacae CRIs in same patient with time interval of 19 days.
