# Supplementary material for: Treatment-Related Toxicities During Anti-GD2 Immunotherapy in High-Risk Neuroblastoma Patients
Source: Front Oncol. 2021 Feb 17;10:601076. doi: 10.3389/fonc.2020.601076 (PMC7925836; doi:10.3389/fonc.2020.601076)
Supplement: Supplementary file 6 [file Table_5.pdf]

Supplementary Table 5: Immunotherapy related toxicities leading to dose modifications of IL-2
Following the indication for the dose modification, is in parentheses the patient number (pt) and the grade (gr) of the indication for dose modification/toxicity. CRI = catheter-related infection; AST = Aspartate transaminase; ALT = Alanine transaminase; AR = allergic reaction;. gr = grade. * Patient 12: received no IL-2 in IT course 4 due to liver transaminase (AST/ALT) abnormalities. ** Patient 26: catheter-related-infection between IT1 and IT2 → no "low dose" IL-2 (day 1 – 4 IT course 2) during IT course 2.

Number of dose modifications	Course 2 n = 26	Indication for dose modification	Course 4 n = 23	Indication for dose modification
0	n = 12		n = 12	
1	n = 7	• AST / ALT (pt9; gr2)<br>• AST / ALT (pt12; gr3)*<br>• Cough (pt13; gr3)<br>• Cough (pt14; gr3)<br>• Cough (pt22; gr3)<br>• CRI (pt3; gr3)<br>• Dyspnea (pt24; gr3)	n = 7	• AR (pt4; gr3)<br>• AST / ALT (pt8; gr3)<br>• AST / ALT (pt17; gr3)<br>• Cough (pt14; gr3)<br>• Creatinine (pt16; gr2)<br>• CRI (pt18; gr3)<br>• CRI (pt21; gr3)
2	n = 4	• AST / ALT (pt21; gr3)<br>CRI (pt21; gr3)<br>• Fever (pt25; gr1)<br>Tachycardia (pt25; gr2)<br>• Hypertension (pt16; gr3) 2x<br>• Pain (pt1; gr4);<br>AR (1; G3)	n = 3	• AST / ALT (pt23; gr3)<br>Hypoxia (pt23; gr3)<br>• Cough (pt25; gr3) 2x<br>• CRI (pt22; gr4)<br>Fever (pt22; gr2)
3	n = 2	• AR (pt7; gr3)<br>Hypotension (pt7; gr3)<br>Fever (pt7; gr2)<br>• CRI (pt26; gr3)**<br>Cough (pt26; gr3)<br>Fever (pt26; gr3)	n = 1	• Cough (pt2; gr2)<br>Cough (pt2; gr3) 2x
4	n = 1	• 4x Cough (pt2; gr2))		
